# Population pharmacokinetic analyses comparing immediate-release levodopa/carbidopa, controlled-release levodopa/carbidopa, and IPX203. Findings from a phase 1 study in healthy volunteers

**DOI:** 10.1007/s00702-026-03233-w

**Published:** 2026-07-21

**Authors:** Achim Wach, Jaakko Kopra, Ivan Marjanovic, Mathias Knecht, Peter Jenner

**Affiliations:** 1 1A Consulting, Dr Wach & Wach GbR, Weil am Rhein, Germany; 2 Zambon Biotech SA, Medical Affairs, Cadempino, Switzerland; 3 Institute of Pharmaceutical Sciences, Faculty of Life Sciences and Medicine, King’s College London, London, SE1 1UL UK

**Keywords:** Carbidopa, IPX203, Levodopa, Population pharmacokinetics, Sinemet

## Abstract

Optimizing oral levodopa delivery by prolonging duration of effect remains a key challenge in managing OFF periods in Parkinson’s disease. IPX203 is an oral modified-release formulation combining immediate-release (IR) and extended-release (ER) levodopa with IR carbidopa, designed to provide rapid onset and sustained exposure. We characterized pharmacokinetics of levodopa and carbidopa following IPX203 compared with IR and controlled-release (CR) levodopa/carbidopa using population pharmacokinetic modeling from a Phase 1 study in healthy volunteers. Plasma concentration–time data were analyzed using nonlinear mixed-effects modeling. Formulation-specific absorption models, including a dual-depot model for IPX203, were combined with a shared systemic disposition model. The models adequately described observed levodopa and carbidopa concentrations across formulations. Marked formulation-dependent differences in levodopa exposure were driven by absorption. IR-levodopa/carbidopa produced rapid absorption with peak concentrations followed by a steep decline, consistent with elimination-rate–limited pharmacokinetics. CR-levodopa/carbidopa showed delayed absorption with lower peak concentrations and limited time within the expected therapeutic range (750–1000 ng/mL). In contrast, IPX203 exhibited biphasic absorption, with an initial IR-driven peak and sustained concentrations from the ER component, consistent with rate-limited absorption and prolonged intestinal residence. This resulted in reduced peak–trough fluctuation and longer duration within therapeutic range. Carbidopa profiles were qualitatively similar, with higher absolute exposure for IPX203. Model-based simulations of commonly used dosing regimens suggested that IPX203 administered every 6 h achieved the most stable levodopa concentrations. These findings indicate that IPX203 provides a distinct exposure profile characterized by rapid onset and sustained levodopa exposure driven by controlled absorption.

**Supplementary Information:**

The online version contains supplementary material available at https://doi.org/10.1007/s00702-026-03233-w.

## Introduction

Immediate-release (IR) forms of levodopa, administered in combination with a peripheral aromatic amino acid decarboxylase inhibitor (AADCi) such as carbidopa or benserazide, remain the cornerstone of symptomatic treatment for Parkinson’s disease (PD) (Olanow and Stocchi 2018). Despite its well-established efficacy, the clinical performance of conventional oral levodopa/AADCi therapy is constrained by pharmacokinetic limitations, including a short plasma half-life, variability in gastric emptying, competition for transport in the proximal small intestine, and substantial dose-to-dose variability in systemic exposure (Demailly et al. 2024). Multiple studies have implicated the resultant ‘pulsatile’ delivery of levodopa using conventional IR formulations in the development and expression of motor complications, including wearing-off and dyskinesias, over the course of the disease (Stocchi et al. 2005; Stocchi 2006; Olanow et al. 2013). All of this complicates the treatment of the early and later stages of PD when motor complications appear, necessitating increases in dose, dosing frequency, or dose fragmentation, which in turn can adversely affect compliance and patient convenience. Ultimately, these limitations have contributed to a growing tendency to resort to device-aided therapies such as intrajejunal levodopa infusion and continuous subcutaneous levodopa delivery, despite their greater complexity, invasiveness, and resource requirements

Overcoming these problems has become a key objective for improving and extending the use of oral levodopa therapy. Pharmacokinetic–pharmacodynamic evaluation has demonstrated that the motor response to levodopa is closely linked to the time that plasma concentrations remain above the threshold for producing a therapeutic response, rather than to peak concentrations alone (Nelson et al. 1990; Stocchi et al. 2005; Nutt 2008; Mao et al. 2013; Mao and Modi 2016; Marsot et al. 2017; Hauser et al. 2018). Plasma levodopa concentration is widely used as a surrogate marker for central dopaminergic exposure, with threshold concentrations in the range of approximately 750–1000 ng/mL associated with the initiation of antiparkinsonian effects, and higher concentrations being associated with peak-related adverse effects (Stocchi et al. 2005, 2010; Marsot et al. 2017). With disease progression, clinical benefit becomes dominated by the short-duration levodopa response, with response fluctuations reflecting the rapid rises and falls in circulating drug levels associated with conventional oral levodopa therapy (Nutt 2008; Stocchi et al. 2010). Conversely, it is now well accepted that maintaining relatively stable plasma concentrations can substantially reduce OFF-time and provide a consistent levodopa therapeutic response (Tang et al. 2025).

IR levodopa/carbidopa (IR-LD/CD) formulations provide rapid absorption but are characterized by high peak plasma drug concentrations followed by a rapid decline, necessitating frequent dosing and contributing to pulsatile dopaminergic stimulation (Nutt 2008; Contin and Martinelli 2010). Controlled-release (CR) oral formulations were developed to prolong exposure; however, delayed, incomplete, and erratic levodopa absorption has limited their ability to reliably sustain plasma concentrations within the therapeutic window (Cedarbaum 1989; Yeh et al. 1989; Contin and Martinelli 2010). As a result, CR formulations are often used as adjunctive therapy, such as at bedtime, rather than as full replacements for IR regimens. To address these limitations, newer oral modified-release (MR) strategies have been designed to better approximate continuous levodopa delivery while retaining the convenience of oral administration. Rather than attempting to replace oral therapy, such approaches aim to reduce the magnitude of the peak–trough oscillations in levodopa levels that are inherent to IR dosing by prolonging the time over which plasma drug concentrations remain within the therapeutic window (di Biase et al. 2023).

IPX203 is an oral MR-LD/CD formulation developed to address the short plasma half-life of IR-LD/CD and the limited window of levodopa absorption in the proximal small intestine (LeWitt et al. 2023). The formulation combines IR levodopa and carbidopa granules to provide a rapid onset of effect with extended-release (ER) pellets designed to maintain levodopa levels for longer. An enteric coating prevents the pellets from disintegrating too early in the stomach and ensures that absorption occurs primarily in the small intestine, while the pellets - formulated using mucoadhesive polymer technology - are designed to remain at this primary site of levodopa absorption for an extended period, helping to sustain drug exposure (LeWitt et al. 2023). Earlier formulations (e.g. IPX066 [Rytary]) demonstrated the feasibility of achieving more sustained systemic levodopa exposure through a combination of IR and ER components (Mittur et al. 2017). By employing a new delivery mechanism designed to prolong residence at the primary site of absorption in the small intestine, IPX203 is intended to further enhance pharmacokinetic consistency and extend the duration of effect. In clinical trials in patients with PD experiencing motor fluctuations, IPX203 was associated with a longer duration of effect per dose and increased daily good ON-time compared with IR-CD/LD, while requiring fewer daily administrations (Hauser et al. 2023, 2025).

Given that, once in the systemic circulation, the downstream distribution, metabolism, and elimination of levodopa and carbidopa are largely formulation-independent, the distinguishing features of IPX203 are expected to arise primarily from its absorption characteristics. However, the extent to which these formulation-specific differences translate into distinct pharmacokinetic profiles relative to existing oral formulations, and how these differences may underpin the observed clinical effects, has not been fully characterized. Population pharmacokinetic (pop PK) modelling can provide quantitative insights into potential differences in exposure profiles by integrating inter-individual variability and sparse sampling, thereby informing hypotheses for subsequent evaluation in clinical studies.

In the present analysis we applied pop PK modelling to data from the Phase 1 crossover study IPX203-B16-05 in healthy volunteers to characterize levodopa and carbidopa pharmacokinetics for IPX203, IR-LD/CD, and CR-LD/CD, to identify formulation-specific differences in absorption profiles, and to use model-based simulations to compare levodopa exposure under commonly used clinical dosing regimens.

## Materials and methods

### Study design and participants

The study was a randomized, single-center, open-label, single-dose, three-treatment, three-period crossover Phase 1 investigation conducted in healthy Asian male volunteers (aged 20–42 years with a body mass index 18.7–29.7 kg/m²). Exclusion criteria included the presence of clinically significant medical conditions, abnormal laboratory or ECG findings, gastrointestinal disorders that could affect drug absorption, a history of drug or alcohol abuse, recent use of prohibited or investigational medications.

Participants were randomized in a 1:1:1 ratio to one of three treatment sequences using a computer-generated randomization schedule; each participant received all three treatments, with dosing periods separated by a six-day washout. Following an overnight fast of ≥ 10 h (abstinence from alcohol for ≥ 48 h), participants received a single oral dose of IPX203 (carbidopa 70 mg/levodopa 280 mg), IR-LD/CD (Sinemet, carbidopa 25 mg/levodopa 100 mg), or CR-LD/CD (Sinemet CR, carbidopa 25 mg/levodopa 100 mg), administered with 240 mL of water. Study drug was swallowed intact, and dosing occurred in the morning under fasting conditions. Participants remained fasted for ≥ 4 h post-dose, after which standardized meals were provided at prespecified times.

Blood samples were collected at predefined time points, processed to plasma under temperature-controlled conditions with stabilizer, and stored frozen until analysis at a central bioanalytical laboratory. Plasma concentrations of carbidopa and levodopa were quantified using a validated liquid chromatography–tandem mass spectrometry (LC-MS/MS) method following automated extraction. Pharmacokinetic parameters were derived using non-compartmental methods based on observed concentration–time profiles.

### Population pharmacokinetic modelling

Pop-PK analyses were performed using non-linear mixed-effects modelling, full details of which are provided in the supplemental appendix. One- and two-compartment structural models with first-order absorption and elimination were explored for levodopa and carbidopa. For IPX203, a dual-depot absorption model was implemented to represent the IR and ER levodopa components, with optional lag time for the ER depot. For Sinemet IR and CR, single-depot absorption models were used.

Inter-individual variability was modelled using exponential random effects, and residual unexplained variability was described using additive, proportional, or combined error models as appropriate. Model selection was based on goodness-of-fit diagnostics, objective function value, and parameter plausibility.

### Model-based simulations

Using the final Pop PK models, levodopa plasma concentration–time profiles were simulated for dosing regimens reflecting common clinical practice, including multiple daily dosing of IR-LD/CD, CR-LD/CD, and IPX203 formulations. Simulated regimens included IR-LD/CD administered at short intervals (e.g., every 2–3 h, with or without a bedtime dose of CR-LD/CD), CR-LD/CD administered at longer intervals (e.g., every 6 h), and IPX203 administered at approximately 5–6-hour intervals at clinically relevant dose levels. Simulations focused on comparative assessment of peak concentrations, trough concentrations, and duration of time within a putative therapeutic range.

## Results

**Fig. 1:**
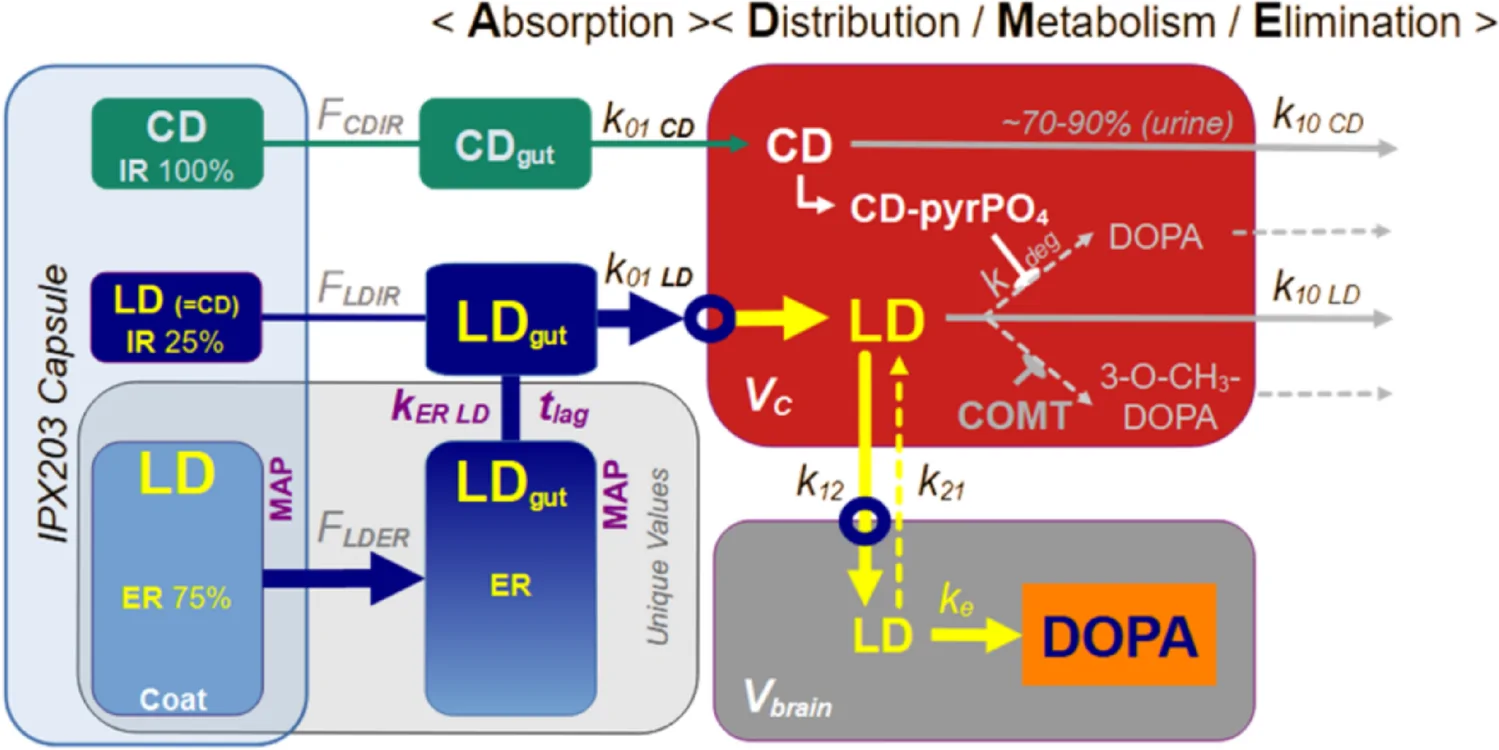
Systemic model for oral LD-CD. *CD* Carbidopa, *LD* Levodopa, *IR* Immediate release component, *ER* Extended release component, *MAP* mucoadhesive polymer, *COMT* Carboxy-methyl transferase, *pyr-PO*_*4*_ pyridoxal phosphate, *F *bioavailability for IR CD (F_CDIR_), IR LD (F_LDIR_), and ER LD (F_LDER_) components; k - rate constants for release from ER (k_ERLD_), absorption (k_01_), LD degradation (kdeg), elimination (k10), intra-compartment transits (i.e., k_12_ and k_21_), and DOPA synthesis (k_e_); *t*_*lag*_ lag time, *V* Volume of central (c) and brain compartment

The final population pharmacokinetic models provided an adequate description of observed levodopa and carbidopa plasma concentration–time data for all three formulations. A common systemic model for oral LD/CD administration was implemented, in which formulation-specific differences were confined to the absorption phase, while distribution, metabolism, and elimination processes were shared across formulations (Fig. 1). Once absorbed into the systemic circulation, levodopa and carbidopa were described using identical downstream disposition parameters irrespective of formulation.

The systemic model incorporated a single absorption depot for IR-LD/CD and Sinemet CR, and two parallel absorption depots for IPX203 to reflect its IR and ER components. For IPX203, the ER levodopa depot was characterized by delayed, rate-limited release consistent with a formulation designed to retain the drug in the upper small intestine for longer, thereby supporting sustained drug uptake. Model diagnostics demonstrated good agreement between observed and population-predicted concentrations, with no evidence of systematic bias across time or concentration ranges.

### Population pharmacokinetic modelling

High-level fitting results with raw data overlays demonstrated that carbidopa and levodopa exposure profiles were well captured by the population pharmacokinetic models across all three formulations. For both analytes, model-predicted concentration–time profiles showed good agreement with observed data, with formulation-specific differences confined to the absorption phase and shared downstream disposition processes.

**Fig. 2:**
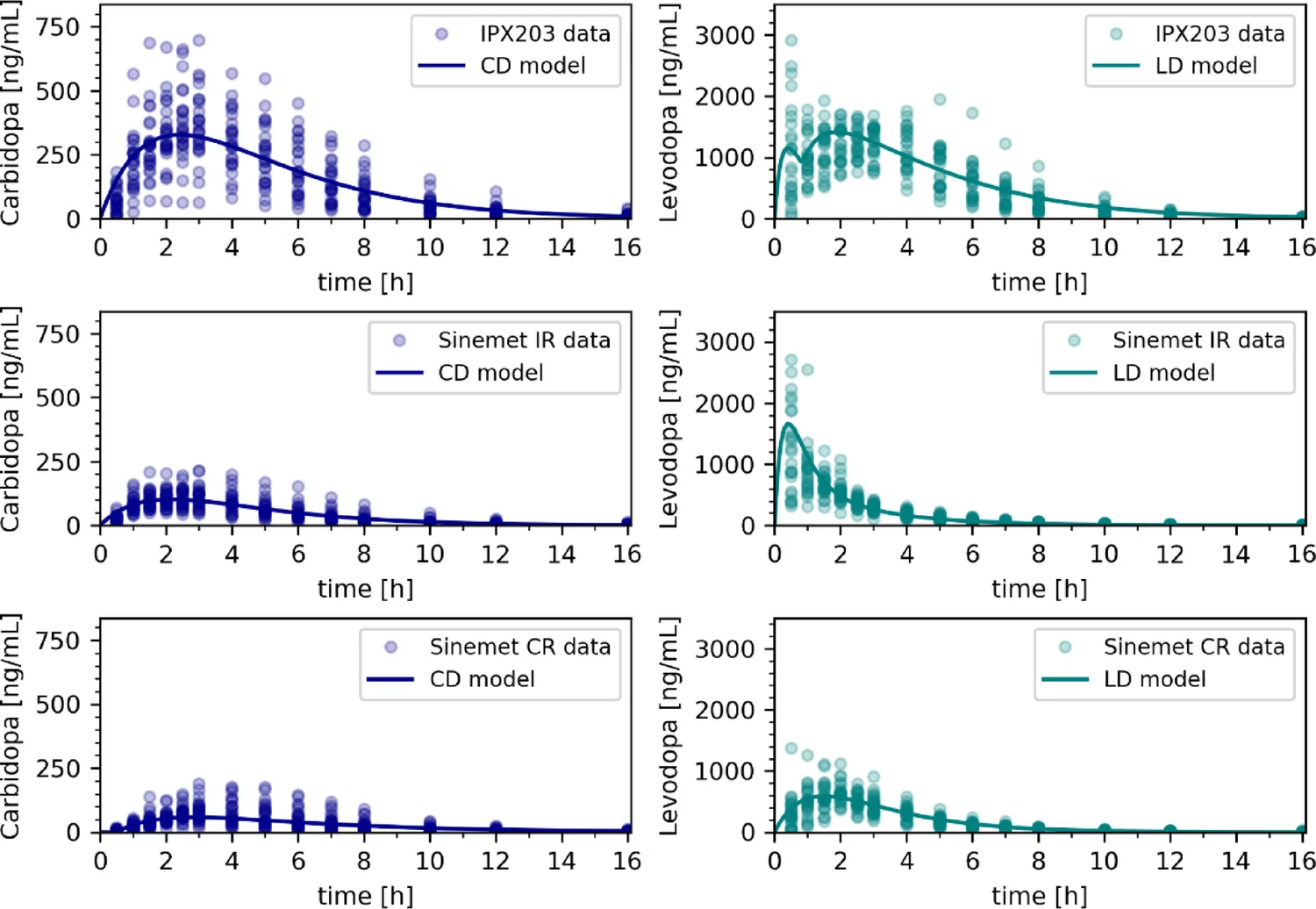
Carbidopa (left) and levodopa (right) plasma exposure in IPX203-B16-05 as a function of time. Symbol color intensities reflect observation frequencies in the entire population. The solid line represents the best-fit model produced by Non-Linear Mixed Effect (NLME) estimation algorithms for each compound

Carbidopa exposure profiles were qualitatively similar across formulations once dose differences were considered. After dose normalization, the temporal shapes of carbidopa concentration–time curves were comparable for IR-LD/CD, Sinemet CR, and IPX203, with peak concentrations typically occurring between approximately 2 and 3 h post-dose (Fig. 2). The higher absolute carbidopa exposure observed with IPX203 reflected the higher administered carbidopa dose (70 mg) relative to IR-LD/CD and Sinemet CR (25 mg each), rather than formulation-specific differences in absorption or disposition. Carbidopa absorption was adequately described using first-order processes for all formulations, and no formulation-dependent differences beyond dose were required to characterize carbidopa distribution or elimination.

In contrast, marked formulation-dependent differences were observed for levodopa, which were already evident in the raw plasma concentration–time data (Fig. 2) and were well described by the models. IR-LD/CD produced rapid levodopa absorption with early peak concentrations at 0.5 h followed by a steep decline, consistent with elimination-rate-limited pharmacokinetics. High inter-individual variability was observed during the early absorption phase, particularly at the first post-dose sampling time. Sinemet CR demonstrated delayed absorption with lower peak concentrations and a limited duration during which levodopa concentrations remained above putative therapeutic thresholds. IPX203 exhibited a biphasic levodopa absorption profile, with an early peak driven by its IR component and sustained concentrations attributable to the ER component. The delayed, rate-limited absorption of levodopa from the ER component was consistent with prolonged residence in the upper small intestine. This resulted in reduced peak–trough fluctuation and a longer duration of levodopa exposure above putative therapeutic thresholds compared with either IR or CR formulations alone.

### Model-based simulations

**Fig. 3:**
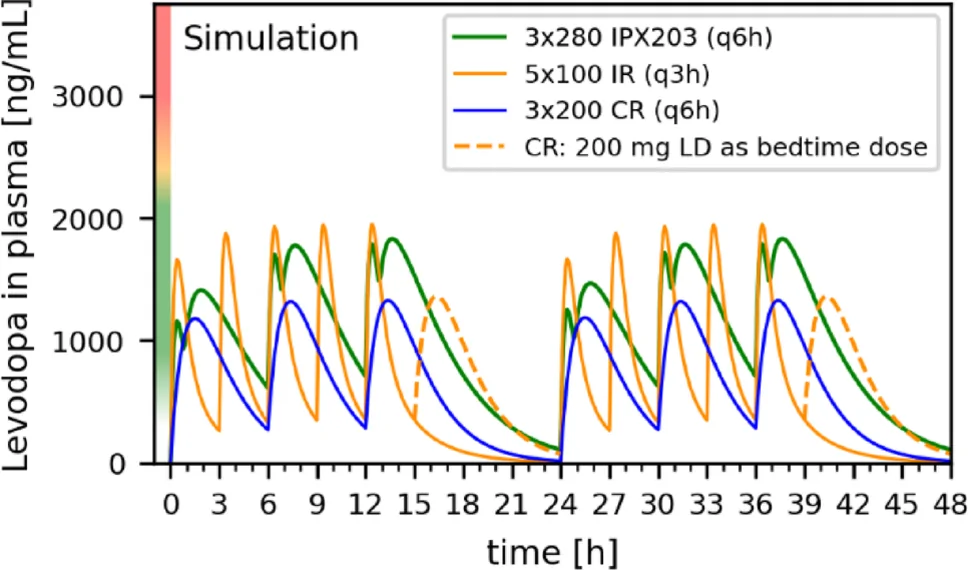
Model based LD exposure estimates for dosing scenarios typically used in clinical practice. Based on published values (Hauser et al. 2018; Mao et al. 2013; Mao and Modi 2016; Nelson et al. 1990), a colour gradient is displayed on the ordinate illustrating the ranges of levodopa plasma concentrations where efficacy is to be expected in PD patients (green zone), as well as indicating ranges (orange and red) where LD-related side effects are likely to occur

The validated models were applied to simulate levodopa exposure under dosing regimens representative of typical clinical use (Fig. 3). Simulations confirmed that IR regimens require frequent dosing to maintain levodopa concentrations within a putative therapeutic range, always at the expense of high peak concentrations and pronounced peak–trough variability. Increasing IR dosing frequency improved the time over which levodopa levels remained above threshold but resulted in peak concentrations approaching or exceeding levels potentially associated with peak-related adverse effects. Simulations further showed that Sinemet CR alone was insufficient to sustain levodopa concentrations within the putative therapeutic range across the dosing interval, even at higher doses, indicating limited usability of CR as a replacement for IR therapy. Combining IR and CR regimens modestly extended time above threshold but increased regimen complexity and did not materially reduce peak–trough fluctuations compared with IR alone. Among the evaluated regimens, IPX203 administered at approximately 6-hour intervals produced the most stable levodopa plasma concentration profiles, maintaining levels above threshold for longer durations while avoiding excessive peak exposure.

## Discussion

Extending the duration of levodopa action and reducing OFF periods remains a central objective in the management of PD, but existing strategies have largely focused on modifying peripheral enzymatic metabolism rather than addressing variability in gastrointestinal absorption. While adjunctive treatments such as COMT, and MAO-B inhibitors can prolong levodopa availability once in the systemic circulation, they do not mitigate the well-recognized variability in levodopa absorption from the gastrointestinal tract.

Against this background, the present analysis provides a quantitative evaluation of how formulation-driven differences in absorption influence levodopa exposure. This Pop PK analysis establishes that differences in levodopa exposure between IPX203, IR-LD/CD, and CR-LD/CD formulations are primarily attributable to formulation-specific absorption characteristics, rather than to differences in downstream disposition processes. A common systemic model adequately described levodopa and carbidopa distribution, metabolism, and elimination across all formulations. These findings support a conceptual separation between absorption-phase effects and post-absorption pharmacokinetics, with formulation-dependent differences arising predominantly from the rate and extent of levodopa absorption

IPX203 can be viewed pharmacokinetically as an IR levodopa input followed by a sustained levodopa input, which is distinct from the delayed and often blunted exposure profile observed with conventional CR formulations. In the present modelling framework, this translated into a shift from predominantly elimination-rate-limited kinetics (typical of IR formulations, with rapid peaks and steep declines) towards absorption-rate-limited kinetics, thereby attenuating peak–trough fluctuations and prolonging the duration for which levodopa concentrations are likely to remain within an efficacious range. By contrast, CR-LD/CD showed delayed and reduced levodopa exposure, consistent with incomplete and/or variable absorption over time, resulting in lower systemic exposure than IR regimens at comparable nominal doses in specific dosing scenarios. These pop PK findings are aligned with prior clinical reports describing the limited clinical utility of CR formulations as monotherapy and the frequent need to supplement CR dosing with IR levodopa in practice (Cedarbaum 1989; Yeh et al. 1989; Stocchi 2006; Contin and Martinelli 2010).

Importantly, these levodopa exposure patterns must be interpreted in the context of concomitant carbidopa delivery. Like most other oral formulations, IPX203 uses a 4:1 levodopa/carbidopa ratio, and the higher absolute carbidopa exposure observed with IPX203 reflects the larger administered dose required to match peak levodopa exposure of the IR comparator, rather than an intrinsic change in carbidopa disposition. Levodopa is routinely co-administered with an AADC inhibitor such as carbidopa to reduce peripheral conversion and increase the fraction available for transport to the brain. Within this framework, the high early systemic carbidopa exposure with IPX203 is mechanistically relevant because carbidopa is delivered exclusively in the IR component, enabling rapid and near-complete peripheral AADC inhibition during the initial phase of levodopa absorption. This effect is driven by formulation design and is proportional to the higher total dose administered; comparable carbidopa exposure would be expected with multiple IR doses providing similar overall levodopa and carbidopa amounts. Taken together, simultaneous Pop PK modelling of levodopa and carbidopa indicates that the differentiated profile of IPX203 observed in clinical practice reflects both sustained levodopa absorption and the timing and magnitude of early AADC inhibition achieved by the IR carbidopa component.

Model-based simulations further indicated that these pharmacokinetic properties may translate into simplified dosing strategies compared with complex IR and IR + CR regimens currently used to approximate more continuous levodopa exposure in clinical practice. Across simulated steady-state scenarios, IPX203 achieved exposure profiles characterized by reduced fluctuation and sustained levodopa concentrations using a single formulation. For example, a single dose of IPX203 produced levodopa exposure comparable in duration to at least two sequential IR administrations, indicating the potential for a substantial reduction in dosing frequency. These findings, however, reflect pharmacokinetic properties rather than clinical effectiveness and should therefore be interpreted within the context of the Phase 1 study population.

Model-derived exposure comparisons provide a quantitative framework for assessing dose relationships between formulations. Consistent with established considerations for conversion from IR to CR levodopa, simulations indicated that achieving exposure profiles with C_max_ values comparable to frequently dosed IR regimens required higher total daily levodopa doses with IPX203 on a milligram basis. This reflects the fact that the additional levodopa content in IPX203 primarily contributes to increased overall exposure (AUC), rather than higher peak concentrations. Across evaluated scenarios, the total daily levodopa dose for IPX203 was ~ 2 times that of IR formulations. In clinical practice, however, conversion to IPX203 is guided by matching C_max_ to the most frequently administered IR dose with a conversion factor of 2.8 rather than by total daily dose equivalence (CREXONT^®^ US Prescribing information, 2026). Accordingly, these ratios represent exposure-based relationships derived from pop PK modelling in healthy volunteers and should not be interpreted as dosing recommendations.

Several limitations should be acknowledged. The analysis relied on single-dose data obtained in healthy, Asian male volunteers, whose demographic characteristics, particularly age, were not reflective of the broader population of patients with PD, thereby limiting external validity. However, there is limited evidence to suggest clinically meaningful differences in levodopa pharmacokinetics between Asian and Western populations, as variability is more strongly driven by factors such as disease stage, dosing, and gastrointestinal absorption. Plasma levodopa concentrations were used as a surrogate for central dopaminergic effects, and the analysis was based on single-dose data rather than the repeated dosing and steady-state exposure that characterize clinical use. Although threshold-based exposure concepts are supported by prior literature, direct pharmacokinetic–pharmacodynamic relationships were not evaluated. Accordingly, extrapolation of these findings to patients with advanced PD should be approached with caution, given the potential impact of altered gastrointestinal transit, disease-related variability, and chronic dosing on levodopa exposure and response. Therefore, these observations should be considered preliminary pending confirmation in patient-based PK/PD analyses, including the planned evaluation of Phase 2 study data.

## Conclusions

Despite these limitations, model-based exposure profiles derived from this Pop-PK analysis were consistent with measured plasma concentration–time data obtained in Phase 1 study participants, supporting the suitability of the systemic model across formulations. The analysis suggests that CR levodopa formulations may provide limited systemic exposure in specific dosing scenarios, potentially contributing to under-exposure relative to IR regimens. IPX203 was characterized by reduced peak–trough fluctuation combined with sustained levodopa supply from its ER components, reflecting a shift toward absorption-rate-limited pharmacokinetics. Compared with available oral levodopa formulations evaluated in this study, IPX203 modelling supports the potential for reduced dosing frequency, with simulated regimens ranging from two to four daily administrations while maintaining stable systemic exposure. Model simulations further indicated that modest variations in IPX203 administration timing had limited impact on overall levodopa exposure profiles, consistent with its modified-release design. Such a pharmacokinetic profile may be relevant for patients with motor fluctuations, in whom maintaining a relatively simple dosing regimen while enabling a more consistent levodopa exposure is often a treatment consideration.

## Supplementary Information

Below is the link to the electronic supplementary material.

Supplementary Material 1

## Data Availability

All data supporting the findings of this study are available within the paper and its Supplementary Information.
