# Supplementary material for: Population pharmacokinetic analyses comparing immediate-release levodopa/carbidopa, controlled-release levodopa/carbidopa, and IPX203. Findings from a phase 1 study in healthy volunteers
Source: J Neural Transm (Vienna). 2026 Jul 21;133(10):2979–86. doi: 10.1007/s00702-026-03233-w (PMC13743624; doi:10.1007/s00702-026-03233-w)
Supplement: Supplementary file 1 [file 702_2026_3233_MOESM1_ESM.docx]

**Supplement**

**Study overview and modelling approach**

Carbidopa (CD) and levodopa (LD) plasma exposures were available from 25 male healthy Asian participants who were randomized to 100 mg Sinemet IR (IR), 100 mg Sinemet CR (CR), and 280 mg IPX203 treatment in a cross-over design in study IPX203-B16-05. Pharmacokinetic (PK) profiles from these individuals were analyzed by treatment formulation applying r-package non-linear mixed effect (NLME) modeling (nlmixr2)^[1]^ using ordinary differential equations to estimate exposure time profiles (**Figure e1**). No allometric parameter scaling was performed during the modeling.

Study participants’ ages ranged from 20-42 years (33.6 ± 6.6). Their body weight was from 50.4–96.1 kg (61.6 ± 10.9), and their BMI was 18.7-29.7 kg/m^2^ (22.6 ± 3.4).

**Figure e1. NLME modeling for each LD/CD formulation.**

Initial parameter values were derived by standard PK techniques from measured IPX203-B16-05 exposure data and cross-checked with literature values were available. Based on these analyses, first-order CD’s absorption rate (k01) initial value was set to k01=0.5 1/h for all three formulations. First-order LD’s absorption rate (k01) from the Sinemet IR tablets was cross-checked with estimates from published Sinemet IR PK data with early post-dose sampling.^[2]^ According to these data, the first order absorption rate for LD from IR estimated by standard PK techniques should be in the range of k01=2.5-10 1/h. For Sinemet CR LD absorption, k01 was initiated at k01=0.5 1/h.

Initial settings for volume of distribution (Vd) was set to standard human body water content (Vd=30-45 L).

CD clearance (CL) was estimated from a terminal half-life of approximately 2.5 h resulting in CL=11 L/h and that for LD was estimated from the typical half-life published for LD in presence of CD: t1/2=1.5 h, resulting in CL=18.5 L/h for LD.^[3]^

Every NLME output was qualified by visual checks plotting individual and population predicted values as a function of measured plasma exposure and by evaluating the goodness of fit from a plot of the population mean exposure profile over the background of measured plasma levels. Both for CD and LD exposure.

CD exposure by Sinemet IR, Sinemet CR, and IPX203 was evaluated in one process block. Afterwards, LD exposure by Sinemet IR and Sinemet CR were modeled followed by NLME modeling of IPX203 based on the best-fit Sinemet IR model.

As depicted in **Figure e1**, the NLME modeling process was started by testing a one compartment model with first-order absorption. This was tested without and with lag time. Next, a standard two compartmental model was applied. Again without and with absorption lag time.

Only for LD exposure analyses of Sinemet IR and CR formulations, in a final NLME run, also time-dependent irreversible inhibition of amino-acid decarboxylase (AADC) by CD co-medication was introduced to the one compartment model. This was done by adding a virtual compartment for AADC driven clearance, which was at steady-state at t=0. CD´s effect was then modeled as a time-dependent irreversible AADC reduction from the virtual compartment based on actual CD concentration in the plasma sample and an inhibitory constant for the CD concentration at which 50% of enzyme inhibition occurs (IC50).

IPX203 modeling was based and restricted to the best-fit model obtained for Sinemet IR. This was a two-compartment model without lag time. For evaluation, the best-fit Sinemet IR NLME model (one absorption depot(IR)) was extended with a second absorption depot(ER). In addition, ER-specific absorption parameters were introduced for depot(ER), namely its first-order absorption rate and its retardation range for absorption delay. The first-order absorption rate from depot(ER) was initiated at a 5-times slower rate than estimated for the IR absorption rate. In addition, a lag-time range of 0.5-3.5 hours was set to allow modeling of an absorption delay from depot(ER).

All other kinetic parameters for formulation independent parameters like central and peripheral volume of distribution (Vc, Vp), distribution (k12, k21), and elimination (CL) of LD were derived and fixed to the values obtained from the best-fit IR model.

Carbidopa exposure was well described by a one compartment structural model for all three tested formulations and NLME output is listed in **Table e1.**

**Table e1:	Pharmacokinetic parameter of carbidopa exposure for IPX203, Sinemet IR, and Sinemet CR formulations**

| **Formulation** | **Parameter** | **Mean (95%CI)** | **BSV (CV%)** |
| --- | --- | --- | --- |
| IPX203 | k_01_ (1/h) | 0.367 (0.324, 0.416) | 25.9 |
|  | Vc (L) | 69.5 (61.3, 78.9) | 26.5 |
|  | CL (L/h) | 32.3 (27, 38.6) | 46.3 |
|  | add Error | 10.0% |  |
| Sinemet IR | k_01_ (1/h) | 0.401 (0.368, 0.438) | 26.8 |
|  | Vc (L) | 78.4 (69.7, 88.2) | 36.4 |
|  | CL (L/h) | 41.3 (35.1, 48.8) | 44.8 |
|  | add Error | 1.31% |  |
| Sinemet CR | k_01_ (1/h) | 0.236 (0.116, 0.479) | 131 |
|  | lag time (h) | 0.493 (0.356, 0.684) | 155 |
|  | Vc (L) | 84.5 | bound (IR) |
|  | CL (L/h) | 58.2 (46.6, 72.7) | 77.5 |
|  | add Error | 1.03% |  |

*CI – confidence interval, BSV – between participant variability, CV% - variability coefficient*

Levodopa exposure was best described by a one compartment structural model for Sinemet CR, but for Sinemet IR and IPX203 a two-compartment model was most suitable. For all three tested formulations, NLME output is listed in **Table e2.**

**Table e2:	Pharmacokinetic parameter of levodopa exposure for IPX203, Sinemet IR, and Sinemet CR formulations**

| Formulation | Parameter | Mean (95%CI) | BSV (CV%) |
| --- | --- | --- | --- |
| IPX203 | *k_01 IR_ (1/h) | 4.03 (2.42, 6.40) | 128 |
|  | *Vc (L) | 34.6 (27.5, 42.6) | 87.4 |
|  | *CL (L/h) | 33.5 (30.1, 36.4) | 54.8 |
|  | *Q (L/h) | 16.4 (10.2, 25.1) | 156 |
|  | *Vp (L) | 19.7 (15.7, 24.6) | 103 |
|  | k_01 ER_ (1/h) | 0.386 | 86.5 |
|  | lag time ER (h) | 0.611 | 79.7 |
|  | add Error | 13.2% |  |
| Sinemet IR | k_01_ (1/h) | 3.94 (2.42, 6.40) | 199. |
|  | Vc (L) | 34.2 (27.5, 42.6) | 81.8 |
|  | CL (L/h) | 33.1 (30.1, 36.4) | 25.0 |
|  | Q (L/h) | 16.0 (10.2, 25.1) | 62.9 |
|  | Vp (L) | 19.7 (15.7, 24.6) | 10.1 |
|  | add Error | 11.9% |  |
| Sinemet CR | k_01_ (1/h) | 0.488 (0.416, 0.572) | 7.08 |
|  | Vc (L) | 45.3 (32.3, 63.5) | 54.0 |
|  | CL (L/h) | 39.6 (35.6, 44.2) | 23.1 |
|  | add Error | 9.87% |  |

**Bound levodopa variability ranges (IR) – initial parameter and lower and upper value boundaries for IPX203 NLME analyses were set as determined for Sinemet IR.*

1. Fidler M, Wilkins JJ, Hooijmaijers R, Post TM, Schoemaker R, Trame MN, Xiong Y, Wang W. Nonlinear Mixed-Effects Model Development and Simulation Using nlmixr and Related R Open-Source Packages. CPT Pharmacometrics Syst Pharmacol. 2019 Sep;8(9):621-633. doi: 10.1002/psp4.12445.
2. Yao HM, Hsu A, Gupta S, Modi NB. Clinical Pharmacokinetics of IPX066: Evaluation of Dose Proportionality and Effect of Food in Healthy Volunteers. Clin Neuropharmacol. 2016 Jan-Feb;39(1):10-7. doi: 10.1097/WNF.0000000000000126.
3. Cedarbaum JM. Clinical pharmacokinetics of anti-parkinsonian drugs. Clin Pharmacokinet 1987;13: 141–178
